# Retrospective comparative analysis of intraocular lens calculation formulas after hyperopic refractive surgery

**DOI:** 10.1371/journal.pone.0224981

**Published:** 2019-11-07

**Authors:** Anibal Francone, Nicole Lemanski, Martin Charles, Sheila Borboli-Gerogiannis, Sherleen Chen, Marie-Claude Robert, Roberto Pineda

**Affiliations:** 1 Centro Oftalmológico Dr. Charles Sociedad Anónima, Buenos Aires, Argentina; 2 Massachusetts Eye and Ear Infirmary, Harvard Medical School, Boston, Massachusetts, United States of America; University of Houston, College of Optometry, UNITED STATES

## Abstract

**Purpose:**

To compare the intraocular lens calculation formulas and evaluate postoperative refractive results of patients with previous hyperopic corneal refractive surgery.

**Design:**

Retrospective, comparative, observational study.

**Setting:**

Massachusetts Eye and Ear Infirmary, Harvard Medical School, Boston, Massachusetts, USA.

**Methods:**

Clinical charts and optical biometric data of 39 eyes from 24 consecutive patients diagnosed with previous hyperopic laser vision correction and cataract surgery were reviewed and analyzed. The Intraocular lens (IOL) power calculation using the Holladay 2 formula (Lenstar) and the American Society of Cataract and Refractive Surgery (ASCRS) Post-Refractive IOL Calculator (version 4.9, 2017) were compared to the actual manifest refractive spherical equivalent (MRSE) following cataract surgery. No pre-Lasik / PRK or post-Lasik / PRK information was used in any of the calculations. The IOL prediction error, the mean IOL prediction error, the median absolute refractive prediction error, and the percentages of eyes within ±0.50 diopter (D) and ±1.00 D of the predicted refraction were calculated.

**Results:**

The Holladay 2 formula produced a mean arithmetic IOL prediction error significantly different from zero (*P* = 0.003). Surprisingly, the mean arithmetic IOL prediction errors generated by Shammas, Haigis-L and Barret True K No History formulas were not significantly different from zero (*P* = 0.14, *P* = 0.49, *P* = 0.81, respectively).There were no significant differences in the median absolute refractive prediction error or percentage of eyes within ± 0.50 D or ± 1.00 D of the predicted refraction between formulas or methods.

**Conclusion:**

In eyes with previous hyperopic LASIK/PRK and no prior data, there were no significant differences in the accuracy of IOL power calculation between the Holladay 2 formula and the ASCRS Post-refractive IOL calculator.

## Introduction

Laser vision correction (LVC), more specifically, Laser assisted in situ Keratomileusis (LASIK) and photorefractive keratectomy (PRK) are popular options for the correction of myopia, astigmatism, and hyperopia. [1–3] Hyperopic LASIK and PRK treatment algorithms are utilized in the treatment of both hyperopia and presbyopia, with and without astigmatism. The uncorrected visual acuity outcomes achieved by laser vision correction in this cohort are outstanding, [4–5] and, in due course for their cataract surgery, post refractive patients expect the same quality of vision achieved by LVC.

However, the calculation of IOL power for cataract surgery after LVC is challenging because the reshaping of the anterior cornea interrupts the normal anterior to posterior curvature ratio, introducing error in the corneal power measurement. [6–7] As described by Haigis and Goes, [8] alterations in the corneal shape are responsible for a radius measurement error, [9] a keratometer index error and an IOL formula error; all of which lead to errors in the measure of the true cornea power, causing a refractive surprise after cataract surgery:hyperopia in post myopic LVC patients and myopia in post hyperopic LVC patients.

The margin of error may be smaller in post hyperopic LVC patients than post-myopic LVC for several reasons. First, hyperopic LVC ablations usually correct a lower amount of hyperopia compared to myopia. [8–9] Second, the central corneal ablation in a myopic treatment creates a more oblate cornea and, in doing so, changes the anterior curvature more centrally than peripherally, reducing the axial length (AL)[10] and the anterior chamber depth (ACD). [11] Hyperopic LVC, in contrast, has fewer changes in these parameters, as the ablation pattern is more of a peripheral ring, in an attempt to create a more prolate cornea. [12]

The internal use of the corneal power as a co-predictor for the effective lens position (ELP) causes the IOL formula error.[13–14] Newer formulas, such as Holladay 2, eliminate the formula error by using seven variables to estimate ELP; Axial length (AL), keratometry (K), horizontal white-to-white (WTW) corneal diameter, pre-cataract refraction (R), ACD, lens thickness (LT) and the age of the patient.[15] Due to the well-documented accuracy and predictability of the Holladay 2 formula,[16–19] we have used it to calculate the IOL power in eyes with previous hyperopic Lasik or PRK, without considering prior-LASIK / PRK data.

The objective of this study was to compare the accuracy and predictability of Holladay 2 formula to those available on the website of the American Society of Cataract and Refractive Surgery (The ASCRS online Post-Refractive IOL Power calculator—version 4.9, 2017).

## Materials and methods

A retrospective chart review of consecutive patients diagnosed with previous hyperopic corneal refractive surgery and cataract surgery between January 1, 2010 and March 2015 in the Cornea Division at the Massachusetts Eye and Ear Infirmary (MEEI),of the Harvard Medical School was performed.

This study was approved by the Institutional Review Board of the MEEI and adhered to the tenets of the Declaration of 133 Helsinki and the Health Insurance Portability and Accountability Act (HIPAA). Cases were identified by a medical billing record search, using the International Statistical Classification of Diseases and Related Health Problems, Ninth Revision (ICD-9) diagnosis code 13.41 for phacoemulsification and aspiration of cataract.

The inclusion and exclusion criteria are summarized in Table 1. Both paper-based and electronic records were reviewed to determine demographic and medical information of all the patients included. Surgical records of patients with previous hyperopic corneal refractive surgery who underwent cataract surgery were analyzed.

**Table 1:** Inclusion and exclusion criteria.

Key Inclusion Criteria	• History of phacoemulsification cataract surgery and primary in-the-bag implantation of posterior chamber intraocular lens • Previous hyperopic corneal refractive surgery (LASIK and/or PRK) • Manifest refractive spherical equivalent (MRSE) performed ≥ 4 weeks after cataract surgery • Post-operative best-corrected visual acuity (BCVA) of 20/32 or better
Key Exclusion Criteria	• Complications from previous refractive surgery. • Inability to achieve secure 'in-the-bag' placement of the IOL (i.e. due to posterior capsule rupture, radial tear in capsulorhexis, vitreous loss, zonular rupture) • Use of corneal sutures • Haptic not in the capsular bag • Decentration of the IOL of more than 1.0 mm • Presence of other ophthalmic pathology causing visual impairment: amblyopia, glaucoma, optic neuropathy, age related macular degeneration, macular edema, retinal detachment, proliferative diabetic retinopathy, ocular inflammation • Corneal opacities or irregularities: previous scarring, dystrophy, ectasia • Corneal astigmatism greater than 1.5 diopters. • Other ocular surgery at time of cataract extractionHistory of YAG laser capsulotomy Uncontrolled diabetes • History of ocular trauma • Any neurological condition which may interfere with performance of required tests.

Best-corrected visual acuity (BCVA) was recorded at each visit, reported in Snellen fraction, and was then converted into logarithm of the minimal angle of resolution (logMAR) values for statistical analysis.

All surgeries were performed by 1 of 3 surgeons (R.P., S.C. and S.B.G.) using a temporal clear corneal incision and phacoemulsification technique with in-the-bag IOL placement. The keratometry (K), axial length (AL), white-to-white (WTW), lens thickness, and anterior chamber depth (ACD) values were obtained with LENSTAR LS900 optical spectroscopy (Haag-Streit, Bern, Switzerland). Keratometry was measured using a 1.3375 index of refraction and the flattest and steepest meridians were included. The TECNIS ZCB00 monofocal IOL (Abbott Medical Optics, California) was implanted. The IOL constant was taken from ULIB webpage [20] and the IOL power was calculated using the Holladay 2 formula. The surgeon selected the IOL power to be implanted based on his or her judgment.

Based on the optical biometric data obtained prior to cataract surgery, the IOL predicted power calculation using the Holladay 2 formula (Lenstar LS900 optical spectroscopy) and the ASCRS Post-Refractive IOL Calculator for Eyes with Prior Hyperopic LASIK/PRK (version 4.9, 2017) was performed and compared to the actual manifest refractive spherical equivalent (MRSE) following cataract surgery. No pre-Lasik / PRK information was used in any of the calculations. As a consequence, we only obtained results from Shammas, [21] Haigis-L [22] and Barret True K No History [23–24] online formulas.

The calculation of the IOL prediction error was based on the work of Wang et al.: [25]

*IOL Prediction Error = Implanted IOL Power—Predicted IOL Power*.

Thus, a positive value indicates that the method predicted an IOL of lower power than the power of the implanted IOL, which would result in post-operative hyperopia. Mean arithmetic and absolute IOL prediction errors, variances in mean arithmetic IOL prediction error, and percentage of eyes within 0.50 diopter (D) and 1.00 D of refractive prediction errors were calculated. Positive mean arithmetic IOL prediction error values indicate that the method underestimated the IOL power; contrarily, negative values indicate that the method overestimated the IOL power. A smaller variance in mean arithmetic IOL prediction error indicates better consistency of IOL prediction with that method. Assuming that 1.00 D of IOL prediction error produces 0.70 D of refractive error at the spectacle plane, [26] the percentage of eyes with a refractive prediction error within ± 0.50 D and within ±1.00 D was computed for each method. Prediction errors were then compared between all methods, and the statistical analysis was performed.

The 1-sample t-test was used to determine whether the mean arithmetic IOL prediction errors produced by various methods were significantly different from zero. Analysis of variance was performed to compare differences in IOL prediction errors between methods. The variances in the mean arithmetic IOL prediction errors were tested using the F-test for variances to assess the consistency of the prediction performance by different methods. The percentage of eyes within certain refractive prediction errors were compared using the chi-square test. The Bonferroni correction was applied for multiple tests. Statistical analysis was performed using Microsoft Office Excel and SPSS for Windows software (version 25, SPSS, Inc.). A p-value less than 0.05 was considered statistically significant.

## Results

**Fig 1:**
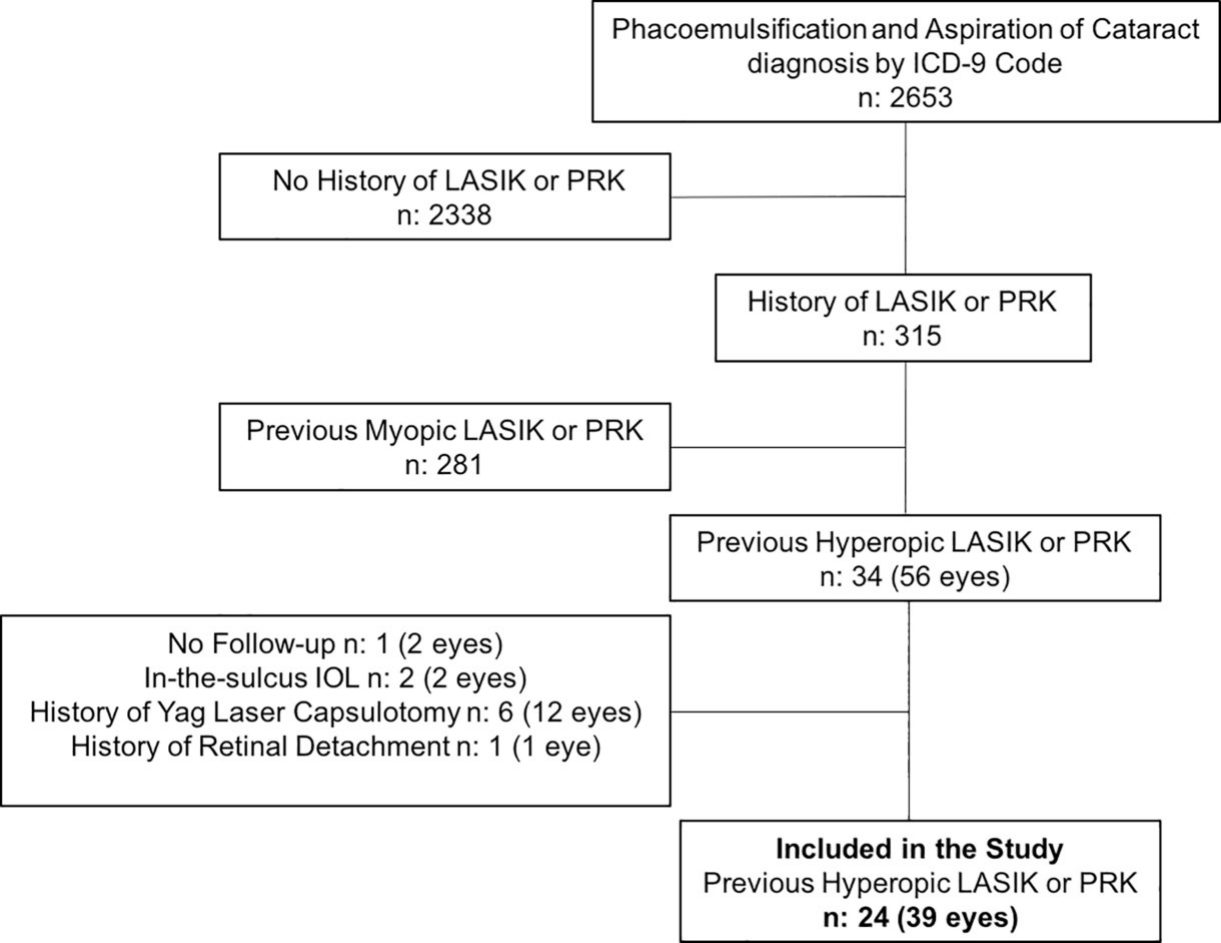
Study design and inclusion of patients during clinical chart review.

The clinical charts of 315 patients diagnosed with a history of LASIK or PRK and cataract surgery were reviewed. 291 patients were excluded because they met one or more exclusion criteria. At the end of the review process, 39 eyes of 24 patients were included in the study (Fig 1). Patient demographics are summarized in Table 2.

**Table 2:** Patient demographics.

Parameter	n*	Mean ± SD	Range
Age (years)	24	67 ± 7	55 to 78
Axial Length (mm)	39	23.3 ± 0.7	22.3 to 24.7
IOL power implanted (D)	39	20.8 ± 1.6	18.0 to 24.5
Post-Cataract MRSE (D)	39	-0.6 ± 0.8	-2.3 to 0.8

IOL = intraocular lens MRSE = manifest refraction spherical equivalent

*Number of eyes except for age, where n is number of patients

**Fig 2:**
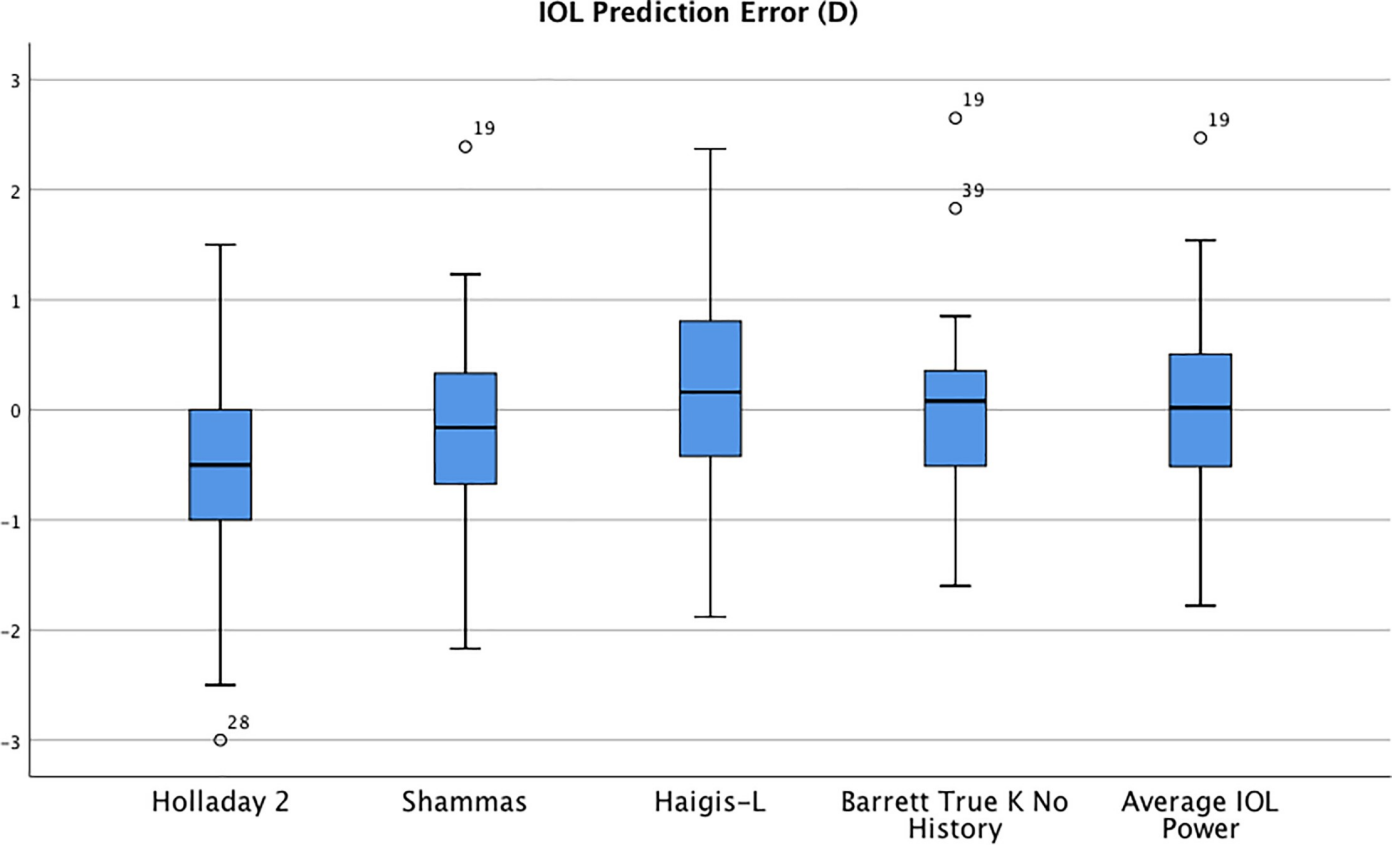
Box Plot of IOL power prediction errors with different IOL power calculation formulas.

Table 3 shows the mean arithmetic and absolute IOL prediction errors. IOL prediction errors including Holladay 2, Shammas, Haigis-L, Barrett True K No History methods and Average IOL power from ASCRS Post-Refractive IOL Calculator are illustrated in Fig 2.

**Table 3:** Mean arithmetic and absolute IOL prediction error using Holladay 2 and no prior data methods from ASCRS IOL calculator (implanted IOL power—predicted IOL power). A positive value indicates that a lower power than the power implanted was predicted and that would result in post-operative hyperopia.

		IOL Prediction Error (D)			
		Arithmetic		Absolute	
Method	Eyes (n)	Mean ± SD	Range	Mean ± SD	Range
LENSTAR					
Holladay 2	39	-0.5 ± 1*	-3 to 1.5	0.7 ± 0.7	0.0 to 2.5
ASCRS					
Shammas	39	-0.2 ± 1	-2.4 to 2.5	0.7 ± 0.7	0.03 to 2.6
Haigis-L	31	0.1 ± 0.9	-1.9 to 2.4	0.7 ± 0.6	0.0 to 2.3
Barrett True K No History	39	-0.03 ± 09	-1.6 to 2.7	0.6 ± 0.6	0.01 to 27
Average (mean) IOL power	39	-0.1 ± 0.9	-2.06 to 2.5	0.7 ± 0.6	0.03 to 2.6

IOL = intraocular lens

*Significantly different from zero (both p< 0.05 with Bonferroni correction)

With no prior data, the mean arithmetic IOL prediction errors were -0.5 D for Holladay 2, -0.2 D for Shammas, 0.12 D for Haigis-L, -0.03 D for Barret True K No History while the mean absolute prediction errorswere 0.69 D, 0.68 D, 0.71 D and 0.64 D, respectively. The Holladay 2 method produced mean arithmetic IOL prediction error that was significantly different from zero (*P* = 0.003). Shammas, Haigis-L and Barret True K No History produced mean arithmetic IOL prediction errors that were not significantly different from zero (*P* = 0.14, *P* = 0.49, *P* = 0.81, respectively). There were no differences in absolute IOL prediction errors between methods in any category. As shown in Table 4, there were not significant differences in variances between methods.

**Table 4:** Variances in arithmetic IOL prediction errors.

Method	Eyes (n)	Variance (D^2^)
LENSTAR		
Holladay 2	39	0.97
ASCRS		
Shammas	39	0.96
Haigis-L	31	0.94
Barrett True K No History	39	0.85
Average (mean) IOL Power	39	0.91

IOL = intraocular lens; D^2^ = square of standard deviation in diopters

Holladay 2 formula had a significantly higher percentage of eyes within ± 0.50 D of the refractive prediction error (71.8%) than methods using no prior data from ASCRS (66.7%, 51.6% and 53.9%, respectively). However, as showed in Table 5, these methods had a higher percentage of eyes within ± 1.00 D of refractive prediction error (84.6%, 83.9%, 89.7%, respectively) than Holladay 2 (79.5%) (all *P* < 0.05 with Bonferroni correction).

**Table 5:** Percentage of eyes within ±0.50 D and ±1.00 D of predicted refraction using various methods. Assuming that 1.0 D of IOL prediction error produces 0.7 D of refractive error at the spectacle plane.

	Percentage	
Method	Within ± 0.50 D	Within ± 1.00 D
LENSTAR		
Holladay 2	71.8	79.5
ASCRS		
Shammas	66.7	84.6
Haigis-L	51.6	83.9
Barrett True K No History	53.9	89.7
Average (mean) IOL Power	66.7	87.2

We were unable to obtain the results of the IOL calculation with Haigis-L formula in eight eyes since the ACD and LT data were not available.

## Discussion

The number of patients undergoing cataract surgery with past history of LASIK or PRK is increasing. [26] The reliability of IOL power prediction formulas in those cases is a problem of clinical significance. There are large, well powered studies evaluating IOL prediction formulas in patients who are post myopic LASIK or PRK,[27–33] but studies evaluating these formulas in patients after hyperopic LASIK or PRK are infrequent and evaluate a small number of patients.[21,34–36] Data pertaining to refraction error, prior to LASIK or PRK, treatment, which would be useful for the clinician and for the IOL power calculation, is often not available. To our knowledge, there are not any studies which have compared formulas that rely on no previous data. The objective of this study was to compare the accuracy of the Holladay 2 formula and the ASCRS Post-Refractive IOL calculator formulas using no prior data.

When performing the statistical analysis, the methods of the ASCRS Post-Refractive IOL calculator produced a mean arithmetic IOL prediction error close to zero, whereas Holladay 2 predicted higher IOL power by 0.5 D. This difference may be due to the fact that Holladay 2 output results are reported in round numbers (0.50 D increments), whereas the predicted IOL power in ASCRS Post-Refractive IOL calculator is given in a continuous numerical scale. Additionally, a mild myopic refractive error is observed, which may be a consequence of the keratometer index error, since we have ignored the change in the ratio between the anterior and posterior cornea after the resulting hyperopic LASIK / PRK that causes an underestimation of effective corneal power and consequently stronger IOL powers.

In accordance with the results of previous studies,[21,25,34] no differences were found regarding the absolute IOL prediction errors between methods. Likewise, the small variance of arithmetic IOL prediction error of each method showed the consistency of IOL prediction performance for each formula.

With the assumption that 1.00 D of IOL prediction error produces 0.70 D of refractive error at the spectacle plane, the absolute prediction error was used to calculate the percentage of eyes within ± 0.50 D and ± 1.00 D of the predicted refraction. Although suboptimal for the current refractive expectations for the patients, in the general population, benchmark standards for refractive outcomes after cataract surgery have been established in the National Health Service of the United Kingdom[37] and the Swedish National Cataract Register Study.[38] These standards are: 55% of eyes achieving refraction within ± 0.50 D of the predicted refraction and 85% of eyes achieving refraction within ± 1.00 D of the predicted refraction. In our study, 2 out of 4 methods (Holladay 2 and Shammas formula) evaluated in eyes with previous hyperopic LASIK/PRK met the benchmark standards set in normal eyes: the formulas Holladay 2 and Shammas met the low benchmark of at least 55% of eyes within ± 0.50 D, the former being the one that reached the highest percentage (71.8%). Only the Barrett True K History formula met the standard of 85% when evaluating the percentage of eyes within ± 1.00 D of the predicted refraction and Holladay 2 had the lowest percentage of the four formulas (79.5%).

The performance of the average (mean) IOL power displayed on the ASCRS Post-Refractive IOL calculator was also evaluated. This method had values that were not significantly different from Holladay 2 and those using no prior data.

This study is the first, to our knowledge, to investigate and compare the post-cataract surgery refractive results of patients with previous hyperopic corneal refractive surgery using the Holladay 2 and the ASCRS Post-Refractive IOL calculator formulas. However, it was a retrospective analysis and the number of patients included is relatively low compared to previous studies evaluating IOL power predictions after myopic LASIK/PRK. Another limitation is that the surgeries were performed by three different experienced surgeons (R.P., S.C. and S.B.G.); nevertheless, in subgroup analysis, there were no statistically significant differences in the mean arithmetic and absolute IOL prediction error values between the three surgeons (*P* = 0.3, *P* = 0.4 and *P* = 0.3). Additionally, ACD and LT data from eight eyes were not available, thus preventing us from using the Haigis-L formula on those eyes. Although Holladay 2 uses LT to estimate ELP, the Holladay IOL Consultant & Surgical Outcomes Assessment Program (HIC.SOAP) discloses that it is not imperative to enter LT value for eyes with AL >22 mm and all those eyes had AL >22 mm. Finally, additional research is required applying existing formulas using Scheimpflug or OCT imaging of the anterior and posterior corneal surface to adjust IOL power after refractive surgery.

In summary, Holladay 2 and methods using no prior data had similar mean absolute IOL prediction errors and variances. Holladay 2 had a greater percentage of eyes within ± 0.50D of the refractive prediction when compared with the ASCRS Post-Refractive IOL Calculator formulas and thus may be used as a guide for IOL power calculation in eyes with previous hyperopic LASIK/PRK if the pre-LASIK/PRK data is not available. The limitations of the current formulas for IOL calculation in eyes with previous hyperopic laser vision correction indicate that further studies are needed.
